# Clinical Characteristics and Outcomes of Pneumocystis jirovecii Pneumonia in Cancer Patients From a Tertiary Care Hospital

**DOI:** 10.7759/cureus.51291

**Published:** 2023-12-29

**Authors:** Muhammad Shehbaz, Seemal Aslam, Muhammad Arslan, Summiya Nizamuddin, Sajid Ali, Salma Abbas

**Affiliations:** 1 Internal Medicine and Infectious Disease, Shaukat Khanum Memorial Cancer Hospital and Research Centre, Lahore, PAK; 2 Microbiology, Shaukat Khanum Memorial Cancer Hospital and Research Centre, Lahore, PAK

**Keywords:** solid organ malignancy, corticosteroids, hematological malignancy, risk factors, pneumocystis jirovecii

## Abstract

Objective

To investigate the predisposing factors, disease course, potential complications, role of primary prophylaxis, and overall outcomes of *Pneumocystis jirovecii *pneumonia (PJP) in cancer patients.

Methods

The study was conducted at Shaukat Khanum Memorial Cancer Hospital and Research Center, Lahore, Pakistan. We analyzed the medical records of cancer patients diagnosed with PJP from January 2018 to December 2022 and collected data about demographic characteristics, clinical presentation, predisposing factors, treatment, complications, and mortality rates. We used SPSS 20 (IBM Corp., Armonk, NY, USA) for data analysis.

Results

Out of 84 patients, 59.5% (n=50) were males and most of the patients belonged to the age group 41 to 65 years. Sixty-seven point nine percent (67.9%; n=57) of patients had underlying hematological malignancy, including three bone marrow transplant recipients while 32.2% (n=27) of patients had underlying solid organ malignancy. We also observed the use of corticosteroids, rituximab, and fludarabine as predisposing factors in 15% (n=13), 27% (n=23), and 3.7%(n=03) of patients, respectively. The most common symptoms were dyspnea (88%; n=74), followed by fever (69%; n=58) and cough (69%; n=58). The former one was more prevalent in hematological malignancy patients as compared to the solid organ tumor group (p-value 0.001). We noted respiratory failure (45.2%; n=38), ICU stay (52.38%; n=44), death (32%; n=27), and shock (10.7% n=9) as the most common PJP-related complications. Moreover, all these complications were more frequent in hematological malignancy patients. We also observed that only three patients developed PJP while on adequate primary prophylaxis for this condition. The overall all-cause one-month mortality was 32% (n=27).

Conclusion

Cancer patients, especially those with hematological malignancies presenting with symptoms suggestive of PJP, need careful evaluation and preemptive treatment as PJP-related mortality is higher in cancer patients. Early diagnosis and treatment in this population can be lifesaving. Moreover, all cancer patients should receive PJP prophylaxis when indicated.

## Introduction

Pneumocystis jirovecii pneumonia (PJP) is an opportunistic, potentially lethal infection of immunocompromised persons. PJP first came into light as a human pathogen in the mid-1940s as an infection of preterm infants and later on in cancer patients in the late 1960s [1]. In the mid-1980s, along with the human immunodeficiency virus (HIV) epidemic, the prevalence of PJP drastically increased, and it emerged as one of the main HIV-related opportunistic infections. However, since the advent of highly effective antiretroviral therapy and chemoprophylaxis for this infection, its incidence among persons living with HIV has been declining in the last two decades. Nowadays, most patients with HIV-associated PJP are treatment naïve, with extremely low CD4 lymphocyte counts, and many of these are not even aware of their HIV status. On the other hand, non-HIV-related PJP cases are increasing with the advent of advanced immunosuppressive treatments like biologics, immune-modulating agents, and stem cell and solid organ transplantations [2].

Cancer is the second most common cause of death worldwide after cardiovascular disorders. The survival rate of cancer patients has significantly increased in the last few decades due to the availability of novel targeted treatment options along with already present conventional therapies. Although the disease progression itself influences the cancer patient’s overall prognosis and survival, infections are also typically associated with significant morbidity and mortality in these immunocompromised patients. Among various infections in cancer patients, PJP has significant importance because of the higher mortality and availability of prophylactic regimens to prevent this infection in high-risk patients [3-5].

Pneumocystis jirovecii pneumonia may develop in cancer or other immunocompromised patients due to the underlying disease or its treatment-related immunosuppression. The well-known predisposing factors for Pneumocystis jirovecii pneumonia in non-HIV patients include hematological malignancies, hematopoietic or solid organ transplantation, the use of certain chemotherapeutic agents for solid organ tumors, and various rheumatic or inflammatory conditions and their treatment modalities (biologics like rituximab and alemtuzumab). Among chemotherapeutic agents, the use of corticosteroids, fludarabine, and temozolomide also poses a significant risk of PJP [5,6].

HIV-related Pneumocystis jirovecii pneumonia has been well-studied, at least in Western countries, and historically, Pneumocystis jirovecii infection has been recognized as an HIV indicator disease. For non-HIV-related PJP, data are sparse, especially from middle to low-income countries with underdeveloped healthcare systems. Moreover, risk assessment for this infection is also complex in non-HIV related patients and cannot be simply determined with CD4 lymphocyte counts as in patients with an HIV infection [6]. Mortality rates are also higher in non-HIV patients as compared to HIV patients (20% to 60% in comparison to 10% to 20%) [6,7].

## Materials and methods

It was a retrospective, single-center, observational study, including polymerase chain reaction-positive Pneumocystis jirovecii pneumonia cases, on a variety of pulmonary specimens (like induced sputum, tracheal aspirate, or bronchoalveolar lavage fluid) from January 2018 to December 2022. We conducted our study at Shaukat Khanum Memorial Cancer Hospital and Research Centre in Lahore, Pakistan, and included both pediatric and adult cancer patients. The Institutional Review Board (IRB) approved the research protocol and waived off written informed consent due to the anonymous and retrospective nature of the study. For all patients, data regarding demographic characteristics, underlying risk factors, clinical features, complications, and outcomes were retrieved from the electronic hospital information system. As our hospital has a fully electronic medical record-keeping facility, after initial data retrieval from the hospital information system, we prepared data sheets keeping patient medical records and names anonymous to avoid any breach of patient confidentiality. We compared the patients with hematological malignancies with solid organ tumor patients with respect to the aforementioned factors to carry out cross-tabulation. We calculated frequencies and percentage proportions for patient demographic characteristics as well as for categorical variables. SPSS 20 was used for data analysis. We applied the chi-square test to calculate the statistically significant association between various outcome variables and underlying predisposing factors. A p-value of less than 0.05 was considered significant.

In our study, we considered the diagnosis of PJP when a patient's medical record fulfilled three criteria, which included a positive real-time PCR for Pneumocystis jirovecii on a pulmonary specimen, the presence of lung infiltrates on chest imaging and clinical signs and symptoms consistent with PJP during their hospitalization. The most commonly used immunosuppressive drugs in cancer patients that predispose to PJP include corticosteroids, fludarabine, rituximab, alemtuzumab, and temozolomide, and in our hospital, only the first three are available so we performed analysis for these three only.

Fever was defined as a temperature of 38 C or higher and dyspnea as shortness of breath of grade 2 or higher as per the Modified Medical Research Council dyspnea scale. (The Modified Medical Research Council scale is a self-rating tool to measure the degree of disability that breathlessness poses on day-to-day activities on a scale from 0 to 4) [6]. Respiratory failure was defined as hypoxia requiring NIV or mechanical ventilation and shock as severe hypotension not responsive to fluid challenge and requiring vasopressors.

As we included all those patients who tested positive for PJP and all of these patients were admitted to the hospital due to severe disease, there may be the possibility that the patient with mild to moderate severity infection did not even bother to consult the hospital. This may be a source of bias in our study.

## Results

Our analysis included 84 patients with confirmed PJP, out of which 59.5% (n=50) were males and most of the patients belonged to the age group of 41 to 65 years. Regarding underlying risk factors predisposing to PJP, 67.9% (n=57) patients had hematological malignancy, including three bone marrow transplantation recipients, and 32.2% (n=27) patients had solid organ malignancy. Among hematological malignancies, patients with Hodgkin lymphoma were most prevalent. Among solid organ malignancies, breast carcinoma patients were the most prevalent in our study. Moreover, we also observed the use of corticosteroids, rituximab, and fludarabine as a predisposing factor in 15% (n=13), 27% (n=23), and 3.7% (n=03) of cases, respectively.

We found dyspnea as the most common presenting symptom (88% (n=78) of patients) followed by fevers (69%; n=58) and cough (69%; n=58). A review of imaging data of our study population revealed that 83.3%(n=70) of patients had bilateral interstitial infiltrates on chest X-ray while the chest X-ray film was unremarkable in just 9.5% (n=08) of patients. The most common computed tomography (CT) chest findings were bilateral, widespread ground-glass opacities, and CT scans of 69% (n=58) patients had these findings. Serum β-d-glucan levels as fungal markers were performed in 31% (n=26) of patients in our study and found to be elevated in 96.12% (n=25) of patients.

Most of the patients received first-line treatment in the form of trimethoprim-sulfamethoxazole at therapeutic doses in oral form (intravenous form was not available with us) while we also noticed that 84% (n=71) of our patients required adjuvant corticosteroids to manage their hypoxemia. We observed respiratory failure (45.2% n=38), ICU stay (52.38%; n=44), death (32%n=27), shock (10.7%n=09), and pneumothorax (7.14%; n=06) as the most common complications. Moreover, all these complications were more frequent in hematological malignancy patients in comparison to solid organ malignancy patients with p-values of 0.06, 0.02, 0.082, 0.26, and 1.0, respectively.

**Figure 1:**
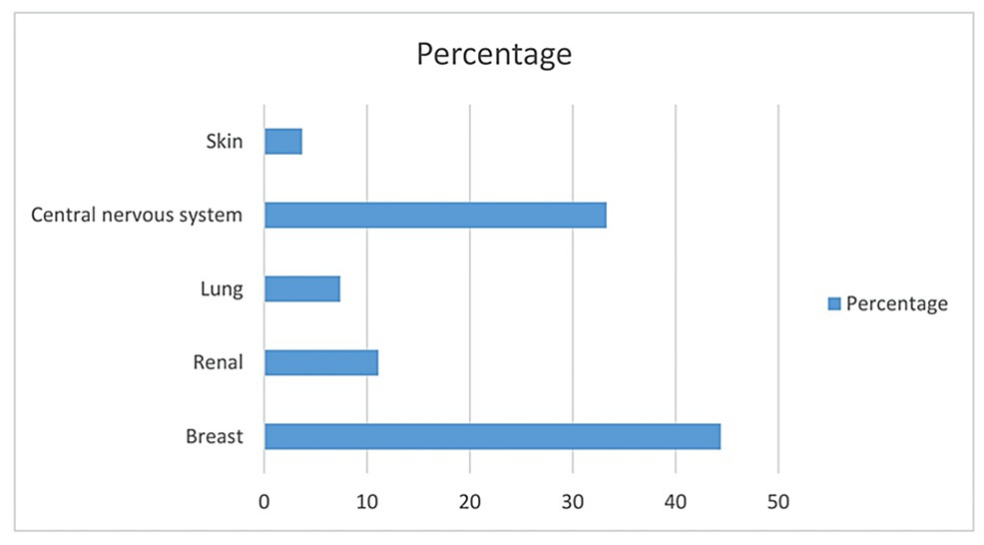
Percentage of various solid tumors in our study population (n=84)

**Figure 2:**
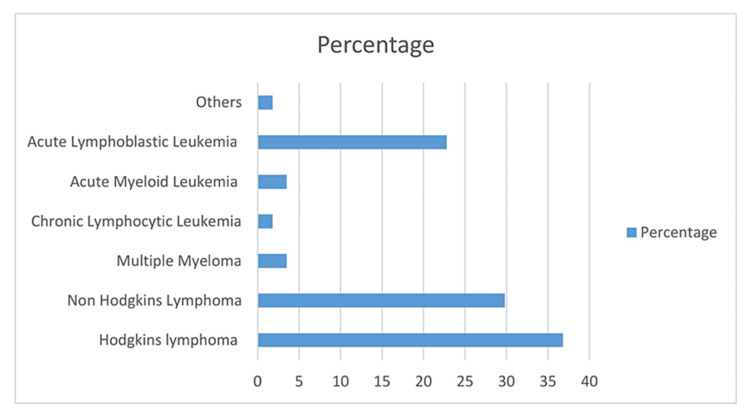
Percentage of various hematological malignancies in our study population (n=84)

One-month all-cause mortality was 32%(n=27) with respiratory failure as the leading cause of death. The mortality rates were higher among hematological malignancy patients in comparison to solid organ tumor patients (38.6%; n=22 in comparison to 18.5%; n=05, p-value .082) (Figure 1). As per our study, risk factors for PJP-related higher mortality in cancer patients include male gender, older age, severe disease requiring mechanical ventilation, and presence of underlying hematological malignancy (Figure 2). We also noticed that only three patients developed PJP while on adequate PJP prophylaxis.

## Discussion

As Pneumocystis jirovecii pneumonia presents with non-specific signs and symptoms, obtaining a thorough history regarding risk factors is pivotal to establish a diagnosis, like a history of HIV infection, any malignancy, or the use of immunosuppressive therapies or high-dose corticosteroids. In our study, we found that the major predisposing factors for PJP infection in cancer patients were hematological malignancies as 68%(n=57) of our patients were of hematological malignancies and this is in accordance with most studies of PJP in cancer patients [8,9]. Moreover, we also noticed high-dose corticosteroids (16%; n=13) and the use of rituximab (27.3%; n=23) as a contributory factor for PJP in cancer patients.

The triad of symptoms (dry cough, progressive dyspnea, and low-grade fever) is well-established for PJP in HIV patients; non-HIV-related PJP cases also present with similar symptoms although the course of illness is more rapid [10]. In our study of cancer patients, we found dyspnea as the most common presenting symptom for PJP (88% n=74) while other common symptoms were fevers (69% n=58) and cough (69%n=58). However, in most of the studies of HIV-related PJP, fever was the most common mode of presentation. This difference regarding clinical presentation in cancer patients may be due to a more widespread and unique type of cancer-related immune paresis. A similar observation was also noted by Eun Hye Lee et al. in a retrospective case-control study of 112 cancer patients with PJP [10,11].

The early diagnosis of PJP is imperative when it comes to its management and disease outcomes. For diagnosis, various imaging and laboratory tests are used. Characteristic findings of PJP on a chest radiograph include bilateral interstitial infiltrates. Chest computed tomography (CT) carries more sensitivity and specificity for diagnosing PJP and ground-glass attenuation is a characteristic finding for PJP on chest CT [12-15]. In our study, a review of imaging data revealed that 83.3%(n=70) of patients had bilateral interstitial infiltrates on chest X-ray, and the most common CT chest findings were bilateral widespread ground-glass opacities, and the CT scans of 69% (n=58) had these findings.

Serum (1, 3)-β-d glucan testing can be useful to assist in the diagnosis of PJP, especially in patients who cannot undergo invasive procedures required for specimen collection and definitive diagnosis. It provides excellent sensitivity, negative predictive value, and negative likelihood ratio of ≥90, ≥97, and<0.12, respectively, allowing PJP to be confidently excluded if negative. (16,17) Serum β-d-glucan positivity alone is not sufficient to confirm the disease because of low specificity. It can give a false-positive result in several scenarios; like the use of cellulose membranes for hemodialysis, the application of glucan-containing gauzes, administration of albumin, intravenous immunoglobulin and blood products, and the use of certain antimicrobials [16-18]. In our analysis, serum (1, 3)-β-d glucan levels as a fungal marker were performed in 31% (n=26) of patients and found elevated in 28.9% (n=25) patients.

The most common complications were ICU stay (52%; n=44), respiratory failure (45%; n=38), death (32%; n=27), shock (11%; n=09), and pneumothorax (7%; n=06). Moreover, we also noticed that these complications were more prevalent in hematology malignancy patients as compared to solid organ malignancy patients (percentages and p-values are given in Table 1).

**Table 1:** Demographic and clinical characteristics of PJP patients with outcomes The data has been represented in the form of percentages (%) and n = number. A p-value less than 0.05 has been considered significant. PJP: Pneumocystis jirovecii pneumonia; TMP-SMX: trimethoprim-sulfamethoxazole

Clinical or demographic characteristic	Total percentage of all patients (n=number)	Percentage in hematological malignancy (n=number)	Percentage in solid organ tumors (n=number)	p-value (<0.05 is significant)
Gender				0.02
Male	59.5%; n=50	71.9%; n=41	33.3%; n=09	
Female	40.5%; n=34	28.07% n=16	66.6%; n=18	
Age				0.087
< 20	21.4%; n=18	29.8%; n=17	3.7%; n=01	
20 - 40	32.1%; n=27	36.84%; n=21	22.2%; n=06	
41 - 65	35.7%; n=30	21.05%; n=12	66.6%; n=18	
> 65	10.7%; n=9	12.28%; n=10	7.4%; n=02	
Steroids use	15.47%; n=13	14.03%; n=08	18.51%; n=05	0.748
Rituximab	27.38%; n=23	40.35%; n=23	0	0.548
Fludarabine	3.57%; n=03	5.26%; n=03	0	
Presentation				
Dyspnea	88%; n=74	98.24%; n=56	66.6%; n=18	0.001
Cough	69%; n=58	66.6%; n=38	74.04%; n=20	0.61
Fever	69%; n=58	71.9%; n=41	62.96%; n=17	0.5
X-ray findings				0.483
Interstitial infiltrates	83.3%; n=70	84.2%; n=48	81.4%; n=22	
Consolidations	06%; n=5	5.2%; n=03	7.4%; n=02	
Pneumothorax	1.2%; n=1	1.7%; n=01	0	
Unremarkable X-ray	9.5%; n=8	10.5%; n=06	7.4%; n=02	
CT findings				0.035
Ground glass opacities	69%; n=58	61%; n=35	85.1%; n=23	
Nodular opacities	2.4%; n=2	3.5%; n=02	0	
Consolidation with nodules	4.8%; n=4	3.5%; n=02	0	
Consolidations	7.1%; n=6	10.5%; n=06	0	
Pneumothorax	1.2%; n=1	1.7%; n=01	0	
CT not done	16.7%; n=14	21%; n=12	7.4%; n=02	
Specimen					0.808
Tracheal aspirate	26.1%; n=22	28.07%; n=16	22.2%; n=06		
Broncho alveolar lavage	65.4%; n=55	63.15%; n=36	70.3%; n=19		
Induced sputum	8.3%; n=7	8.7%; n=05	7.4%; n=02		
Treatment				0.535
TMP-SMX	16%; n=13	17.54%; n=10	11.1%; n=03	
TMP-SMX and Steroids	84%; n=71	82.45%; n=47	88.88%; n=24	
Complications				
Respiratory failure	45.2%; n=38	52.6%; n=30	29.6%; n=08	0.06
Shock	10.07%; n= 09	14.03%; n=08	3.7%; n=01	0.26
ICU stay	52.38%; n=44	61.4%; n=35	33.3%; n=09	0.02
Pneumothorax	07.14%; n=06	07.01%; n=04	7.2%; n=02	1
Overall outcome				
Recovery	68%; n=57	64.91%; n=37	74.07%; n=20	0.082
Death	32%; n=27	38.59%; n=22	18.51%; n=05	

One-month all-cause mortality was 32% (n=27), and this indicates higher PJP-related mortality in cancer patients as compared to HIV patients. Ali Bin Sarwar Zubairi et al. and many others also concluded with similar results [18]. Furthermore, in another study of non-HIV-related PJP patients, Bollée G et al. noted an overall mortality rate of 30%, which is higher as compared to HIV patients. In a meta-analysis of 13 studies including 867 non-HIV-related PJP cases, Professor Yao Liu et al. also concluded an overall PJP-related mortality of 30.6% [19-21].

Since the mortality is higher in cancer patients, a rigorous prophylactic regimen is mandatory when indicated. It is recommended that chemoprophylaxis for PJP be started in non-HIV patients with absolute peripheral lymphopenia, with the use of high doses of corticosteroids (20 mg or higher of prednisolone for a period of one month or higher) with or without other immunosuppressive agents, patients with lymphoma being treated with rituximab-containing regimens, allogeneic hematopoietic stem cell transplantation (HSCT) patients for at least six months post-transplant, can also be used in autologous HSCT patients for three to six months after transplant. Acute leukemia patients should receive PJP prophylaxis throughout anti-leukemic therapy [13,14]. Moreover, our study also underlines the importance of PJP prophylaxis by revealing that only three cancer patients developed this infection while on adequate prophylaxis [20,21].

Our study has some limitations as well, like its retrospective single-center design and relatively small sample size. We were also unable to take a history from patients in real-time regarding clinical presentation, and the data were collected by reviewing patients’ charts in the hospital information system.

Despite its limitations, our study findings will contribute significantly to literature regarding predisposing factors, clinical presentation, complications, and outcomes of PJP in cancer patients from low to middle socioeconomic settings providing a basis for early diagnosis and preemptive treatment for this less common, rather rare type of infection in cancer patients.

## Conclusions

PJP is a serious infection of the lungs that can result in death if not treated in a timely fashion. The results of our study suggest that cancer patients, specifically patients with hematological malignancies with symptoms suggestive of pulmonary infection should be carefully evaluated for PJP infection. Early diagnosis and treatment can be lifesaving. Moreover, all cancer patients should receive PJP prophylaxis when indicated as per the National Comprehensive Cancer Network NCCN Clinical Practice Guidelines in Oncology Prevention and Treatment of Cancer-Related Infections.
